# Evaluation of IFNAR2 and TYK2 transcripts’ prognostic role in COVID-19 patients: a retrospective study

**DOI:** 10.3389/fcimb.2024.1356542

**Published:** 2024-04-29

**Authors:** Alireza Razavi, Maedeh Raei, Yasin Hatami, Ghazal Saghi Chokami, Yasaman Goudarzi, Roya Ghasemian, Reza Alizadeh-Navaei, Hossein Yarmohammadi, Masood Soltanipur, Mohammad Tabarestani, Reza Valadan, Faranak Meshkinfam Haghighi, Abbas Khonakdar Tarsi, Bahar Razavi

**Affiliations:** ^1^ Student Research Committee, School of Medicine, Mazandaran University of Medical Sciences, Sari, Iran; ^2^ Research Center for Clinical Virology, Tehran University of Medical Science, Tehran, Iran; ^3^ Gastrointestinal Cancer Research Center, Non-Communicable Diseases Institute, Mazandaran University of Medical Sciences, Sari, Iran; ^4^ Central Human Immunodeficiency Virus Laboratory, Mazandaran University of Medical Sciences, Sari, Iran; ^5^ Antimicrobial Resistance Research Center, Department of Infectious Diseases, School of Medicine, Mazandaran University of Medical Sciences, Sari, Iran; ^6^ Medical Students Research Committee, Shahed University, Tehran, Iran; ^7^ Molecular and Cell Biology Research Center, Faculty of Medicine, Mazandaran University of Medical Sciences, Sari, Iran; ^8^ Department of Immunology, Faculty of Medicine, Mazandaran University of Medical Sciences, Sari, Iran; ^9^ Department of Psychiatry, Roozbeh Hospital, Tehran University of Medical Sciences, Tehran, Iran; ^10^ Department of Clinical Biochemistry and Genetics, Faculty of Medicine, Mazandaran University of Medical Sciences, Sari, Iran; ^11^ Medical Research Center, Islamic Azad University, Tehran, Iran

**Keywords:** coronavirus disease 2019, interferon alpha-beta receptor IFNAR2 subunit, prognostic factor, SARS coronavirus 2, tyrosine kinase

## Abstract

**Background and objectives:**

This study aimed to investigate the possible prognostic significance of interferon alpha–beta receptor subunit 2 (IFNAR2) and tyrosine kinase 2 (TYK2) expressions.

**Methods:**

We conducted a retrospective study including COVID-19 adult patients. All blood samples were collected before any interventions. The expressions of IFNAR2 and TYK2 were assessed using real-time PCR in venous blood samples of 54 cases and 56 controls. The transcript quantities of IFNAR2 and TYK2 genes were assessed using a Delta-Ct method.

**Results:**

Our findings show no significant differences in gene expression levels for IFNAR2 and TYK2 between patients who required oxygen (O2) therapy and those who did not (p-value = 0.732 and p-value = 0.629, respectively). Likewise, there were no significant differences in IFNAR2 and TYK2 expressions between patients hospitalized for less than 7 days and those hospitalized for 7 days or more (p-value = 0.455 and p-value = 0.626, respectively). We also observed a weak correlation between IFNAR2 expression and CRP (p-value = 0.045, r = 0.192). There was a negative correlation between the expression levels of IFNAR2 and TYK2 transcripts in COVID-19 patients (p-value = 0.044; partial correlation coefficient = -0.283). Additionally, IFNAR2 and TYK2 were significantly downregulated in the COVID-19 group compared to healthy subjects (p-value = 0.002 and p-value = 0.028, respectively). However, neither IFNAR2 nor TYK2 expression was significantly different between the case subgroups based on COVID-19 severity. The IFNAR2 ΔΔCt (B = -0.184, 95% CI: -0.524–0.157, p-value = 0.275) and the TYK2 ΔΔCt (B = 0.114, 95% CI: -0.268–0.496, p-value = 0.543) were not found to be significant predictors of hospitalization duration. The area under the curve (AUC) for IFNAR2 expression is 0.655 (p-value = 0.005, 95% CI: 0.554–0.757), suggesting its poor discriminative value.

**Conclusion:**

We were unable to comment definitively on the prognostic power of IFNAR2 and TYK2 expressions in COVID-19 patients, and larger-scale studies are needed. The principal limitations of this study included the lack of longitudinal analysis and limited sample size.

## 1 Introduction

It is justifiable to assert that the coronavirus disease 2019 (COVID-19), caused by severe acute respiratory syndrome coronavirus 2 (SARS-CoV-2), is currently regarded as a substantial global health threat in the 21st century (Rahimi and Abadi, 2020). SARS-CoV-2 leads to a diverse spectrum of clinical outcomes (Kaser, 2020). The development of severe COVID-19 in an infected individual is determined by intricate interactions between host and viral factors (Shojaei et al., 2023). Finding trustworthy biomarkers to forecast disease outcomes in COVID-19 patients has attracted serious attention since the onset of the COVID-19 pandemic. These biomarkers facilitate the prioritization of resources and the triage of patients, both of which are critical when healthcare facilities are faced with the challenge of treating a substantial volume of COVID-19 patients simultaneously (Shojaei et al., 2023). Multiple prognostic indicators have been proposed for COVID-19, including demographic factors, comorbidities, lung computed tomography (CT) findings, coagulation status, white blood cell counts, and inflammatory response biomarkers, such as C-reactive protein (CRP) and cytokines (Gallo Marin et al., 2021). However, these studies have not yet provided a complete understanding of why some patients experience mild or asymptomatic cases of COVID-19 while others develop severe symptoms and, tragically, lose their lives (Zeberg and Pääbo, 2020). In addition to these predisposing factors for the severe form of COVID-19, genetic factors can also play a role (Zabihi Rizi et al., 2023).

A tyrosine kinase (TYK) belonging to the Janus Kinase family (JAK) is encoded by the TYK2 gene. These kinases play critical roles in regulating the immune system and cell growth (Wallweber et al., 2014). The binding sites for type I IFN are interferon-α/β receptors 1 and 2 (IFNAR1 and IFNAR2, respectively), which are linked to the Janus Kinases TYK2 and JAK1, respectively. IFN-stimulated gene factor 3 (ISGF3) transcription factor and IRF-9, a member of the IRF family, form a heterotrimer as a result of the activation of these kinases, which results in the tyrosine phosphorylation of STAT1 and STAT2 (Yaugel-Novoa et al., 2023).

The root cause of respiratory failure in SARS-CoV-2 patients is exacerbated lung inflammation brought on by dysregulated cytokines. IFN (IFN-I and IFN-II) production was impeded in COVID-19 patients with severe illness, and IFN-stimulated genes were downregulated (Blanco-Melo et al., 2020; Hadjadj et al., 2020). Pattern recognition receptors (PRRs), including the Toll-like receptors (TLR3, TLR7, and TLR8), retinoic acid-inducible gene 1 (RIG-1), melanoma differentiation-associated protein (MDA5), and protein kinase C (PKC), interact with virus-associated molecular patterns when the virus interacts with the host cells (Kawasaki and Kawai, 2014). Triggering these PRRs leads to the initiation of cellular signaling pathways that ultimately culminate in the production of IFN-I (Fitzgerald et al., 2003; Thiel and Weber, 2008).

Some evidence showed that there was no difference in IFNAR1 concentrations between groups isolated by the severity of COVID-19, although IFNAR1 levels appeared to be inversely associated with the severity of COVID-19 (Zhang et al., 2022; Yaugel-Novoa et al., 2023). A separate study reported that higher levels of the soluble receptor (sIFNAR2) were observed in survivors of COVID-19 compared to the group of non-survivors, which was not associated with the scrutinized IFNAR2 genetic variants (Fricke-Galindo et al., 2022). According to recent research, severe instances of COVID-19 were associated with decreased expression of IFNAR2 at 21q22.1 and increased expression of a gene near TYK2 at 19p13.2 (Pairo-Castineira et al., 2021).

In individuals with severe or critical COVID-19, a functional interaction of the TYK2 receptor subunit with the suppressor of cytokine signaling 1 (SOCS1) and SOCS3 has also been documented (Johnson et al., 2020; Low et al., 2022). A study demonstrated that the expression of the TYK2 gene was reduced in COVID-19 patients compared to healthy individuals (Akbari et al., 2022). Furthermore, TYK2 SNPs may be used to determine high-risk individuals for severe COVID-19 (Zabihi Rizi et al., 2023). Interestingly, the presence of some alleles (T in rs2304255, A in rs12720354, and G in rs12720207) of TYK2 decreases its expression and may predict COVID-19 severity (Zabihi Rizi et al., 2023).

Nevertheless, the potential of IFNAR2 and TYK2 expressions as prognostic biomarkers for COVID-19 has yet to be fully established. Comprehensive research in this area holds the promise of shedding light on the molecular mechanisms underlying the severe and critical forms of COVID-19. This knowledge could subsequently be leveraged for therapeutic purposes to enhance patient survival rates (Baillie, 2014). Through the conduct of these studies, we aim to make a valuable contribution to ongoing biomedical research endeavors aimed at combating SARS-CoV-2. Additionally, our findings can guide current genetic and genomic research, facilitating further exploration of candidate gene variants that may assist in stratifying individuals based on their risk levels. In this retrospective research, we delve into the prognostic role of IFNAR2 and TYK2 expressions in the progression of COVID-19 disease.

## 2 Methods

### 2.1 Study design and participants

We conducted a retrospective study involving 56 adult patients diagnosed with COVID-19, who were referred to Imam Khomeini Hospital in Sari in 2022. The diagnosis was confirmed by assessing nasopharyngeal swab samples. At the time of admission to triage, peripheral blood was collected from patients who had a positive reverse transcription-polymerase chain reaction (RT-PCR) test for SARS-CoV-2. All blood samples were collected before any interventions. Patients with a history of hypertension, autoimmune diseases, malignancies, asthma, chronic kidney disease, any congenital immune system defects, liver cirrhosis, hepatitis, hepatitis vaccination, and those using medications that affect IFNAR2 and TYK2 genes, were excluded. Control specimens were obtained in equal quantities from unaffected individuals without any underlying disorders. The study protocol received approval from the Ethical Committee of Mazandaran University of Medical Sciences (IR.MAZUMS.REC.1400.10406). Informed consent was acquired from all patients and controls. Laboratory parameters were collected from all patients. The sample size for both groups, comprising 56 individuals with COVID-19 and 56 controls, was determined based on an α = 0.05, a 90% power, and β = 0.1 and p1 = 0.34 and p2 = 0.66 (according to the risk allele frequency reported by Pairo-Castineira et al. (2021) for the IFNAR2) by STATA software (Version 12.0; Stata Corporation, Lakeway, TX, USA).

### 2.2 Definition of COVID-19 severity

We adopted a modified version of the COVID-19 severity definition provided by the CDC (https://www.covid19treatmentguidelines.nih.gov/overview/clinical-spectrum). In this revised definition, COVID-19 disease is characterized by the presence of symptoms typically associated with COVID-19, such as fever and sore throat, along with a positive SARS-CoV-2 detection using a virus nucleic acid amplification assay (qPCR). A patient with confirmed COVID-19 (as diagnosed by the admitting clinician) who did not require hospitalization was classified as having a mild disease. ‘Moderate’ disease was defined as the need for hospitalization due to COVID-19, and ‘severe’ disease was characterized by the necessity of mechanical ventilation in an intensive care unit (Shojaei et al., 2023).

### 2.3 Experiments

**Figure 1:**
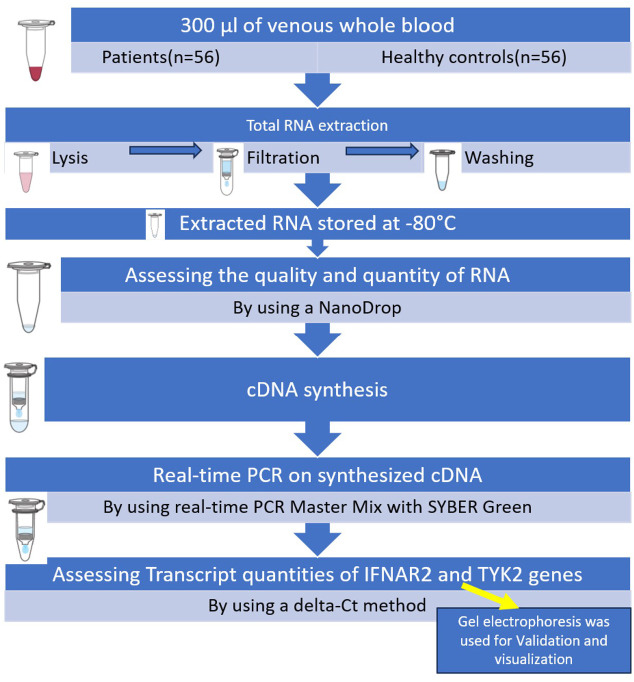
Summary of the steps of performing the executive method in the evaluation of IFNAR2 and TYK2 gene expressions.

Two samples of 2 mL of venous whole blood in EDTA tubes (Liuyang SANLI Medical Technology Development Co, LTD, Liuyang, China) were obtained from patients who had the RT-PCR test for SARS-CoV-2 at the time of admission to triage. Blood samples were collected prior to any interventions. Finally, 300 μL of venous whole blood was utilized from all patients and healthy controls. Subsequently, total RNA was extracted from whole-blood samples using the Total RNA mini kit purification (FAVORGEN Biotech Co, Taiwan) in three primary steps: lysis, filtration, and washing following the manufacturer’s instructions. The extracted RNA samples (extracted from white blood cells and platelets) were then stored at −80°C. A NanoDrop1000 spectrophotometer (Biochrom, Cambridge, UK) was used to assess the quality and quantity of RNA. The purity of RNA was determined by comparing the absorbance at 260 nm and 280 nm. In general, RNA is considered to be “pure” with a ratio of around 2.0. Subsequently, complementary DNA (cDNA) was reverse-transcribed from these DNA-free RNA using the Yektatajhiz cDNA synthesis kit (Iran) based on the manufacturer’s instructions. Real-time PCR was conducted using real-time PCR Master Mix with SYBER Green (Amplicon, Odense, Denmark) on synthesized cDNA, IFNAR2 (FW: ATGATTCGCCTGATTACACA; RV: ACAGTTCTTAACCACCTTCAA), and TYK2 (FW: CCATCATTCCGCACCATC; RV: GGATCGTAGCAGTACAAGC). GAPDH (FW: GTCTCCTCTGACTTCAACAGCG; RV: ACCACCCTGTTGCTGTAGCCAA) was utilized as the internal control (**Figure 1**). Transcript quantities of IFNAR2 and TYK2 genes were assessed using a Delta-Ct (ΔCt) method. To validate and visualize the results obtained from real-time PCR, gel electrophoresis (Bio-Rad, Hercules, CA, USA) was used as detailed in the **Supplementary Methods**.

### 2.4 Statistical analysis

Descriptive statistics were employed to summarize demographic and clinical characteristics. For continuous variables, normality was assessed using the One-Sample Kolmogorov–Smirnov. The results were reported as either mean ± standard deviation (SD) when normality assumptions were met, and median ± interquartile range (IQR) if normality assumptions were violated. Pearson’s correlation coefficient (r) was used to measure the statistical association between variables. The interpretation of Pearson’s correlation coefficients was informed by the study conducted by Chan et al. (Chan, 2003). If needed, partial correlation was used to eliminate confounding effects. One-way ANOVA or a two-tailed Student’s *t*-test was used to analyze data when they exhibited a normal distribution. Alternatively, when the data did not follow a normal distribution, the Kruskal–Wallis test was utilized. Fisher’s exact test was employed to ascertain non-random associations between two categorical variables. Linear regression analysis was applied to model the relationship between a dependent variable and multiple independent variables, aiming to predict the outcome of the dependent variable. K-means clustering analysis was utilized to group similar data points in a process known as clustering. To evaluate the diagnostic efficacy of IFNAR2 ΔΔCt and TYK2 ΔΔCt (IFNAR2 and TYK2 expressions), we conducted a receiver operating characteristic (ROC) curve analysis. All statistical analyses were carried out using SPSS version 20 (SPSS Inc., version 20, Chicago, IL, USA), with a significance level set at 0.05.

## 3 Results

Our cohort comprised 56 control subjects, accounting for 50.9% of the total, and 54 cases, representing 49.1%. Two patients were excluded from the analysis (one due to being in the critical group and another in the asymptomatic group in terms of the severity of COVID-19). Among the cases, they were further categorized into mild (*n* = 22, 20.0%), moderate (*n* = 21, 19.1%), and severe (*n* = 11, 10.0%) subgroups. No statistically significant differences were detected among the groups regarding the distribution of sex, with 52 females (47.3%) and 58 males (52.7%) (**Table 1**, *p* = 0.840), or in terms of age, with means of 42.30 ± 17.70 and 46.22 ± 13.88, respectively, (**Table 1**, *p*-value = 0.200). The BMI values for the mild, moderate, and severe subgroups were 28.19 ± 5.46, 28.62 ± 4.19, and 29.11 ± 6.71, respectively. Importantly, there were no significant differences observed among these subgroups (*p* = 0.895). Among the 23 patients (20.9%), a history of COVID-19 was reported. The mean interval between the last vaccine dose and sample collection was 105.75 ± 80.568 days, with a range of 10 to 365 days. Among the subjects, 91 (82.7%) had a positive vaccination status, with Sinopharm being the most prevalent vaccine type (*n* = 54, 58.9%). A total of 85 individuals (93.4%) had received more than one vaccine dose. Comorbidities were present in 22 patients (40.7%), including hypothyroidism (*n* = 8, 14.8%), hyperlipidemia (*n* = 3, 5.6%), diabetes (*n* = 4, 7.4%), cardiovascular diseases (*n* = 3, 5.6%), and other types (*n* = 3). The CT scans showed normal results in 22 patients, while lung involvement was as follows: <25% in 8 patients (15.4%), 25–50% in 19 patients (36.5%), and >50% in 3 patients (5.8%).

**Table 1:** Demographics, history of vaccination, COVID-19 signs and symptoms, and treatments.

		All participants (*n* = 110)			*p*-Value
		Control (*n* = 56)	Case (*n* = 54)		
Demographics					
**Age(year) ^1^**		42.30 ± 17.70	46.22 ± 13.88		0.200 ^2^
**Sex ^3^**	Female	27 (48.2)	25 (46.3)		0.840 ^4^
	Male	29 (51.8)	29 (53.7)		
		*Mild*	*Moderate*	*Severe*	
**BMI**		28.19 ± 5.46	28.62 ± 4.19	29.11 ± 6.71	0.895 ^5^
History of Vaccination and COVID-19					
		Control (*n* =56)	Case (*n* = 54)	Total (n = 110)	
**Vaccinated patients ^3^**		53 (94.6)	38 (70.3)	91(82.7)	
**Vaccine doses ^3^**	One	1 (1.8)	5 (13.1)	6 (6.5)	
	Two	17 (32.0)	28 (73.6)	45 (49.4)	
	Three	35 (66.0)	5 (13.1)	40 (43.9)	
**The gap between the last vaccine dose and sample collection (Day) ^6^**		86.29 ± 44.57	130.25 ± 104.48	105.75 ± 80.568, (10,365)	
**Vaccine type ^3^**	AstraZeneca	14 (26.4)	6 (15.7)	20 (22.2)	
	Sinopharm	29 (54.7)	25 (65.7)	54 (58.9)	
	Sputnik v	4 (7.5)	0	4 (4.4)	
	Bharat	6 (11.3)	0	6 (6.7)	
	multiple	0	7 (18.4)	7 (7.8)	
**History of COVID-19 ^3^**		13 (23.2)	10 (18.5)	23 (20.9)	
Signs and Symptoms					
**Fever ^3^**		36 (66.7)			
**Cough ^3^**		32 (59.3)			
**Myalgia ^3^**		20 (37.0)			
**Chills ^3^**		28 (51.9)			
**Systolic Blood pressure ^6^**		117.18 ± 27.08, (24.8, 163.0)			
**Diastolic Blood pressure ^6^**		77.23 ± 15.085, (40,101)			
**Temperature ^6^**		36.97 ± 0.5849, (36.0, 38.7)			
**Heart Rate ^6^**		89.21 ± 16.29, (60,130)			
**CT scan results** **(Percentage of pulmonary involvement) ^3^**	Normal	22(42.3)			
	<25	8(15.4)			
	25–50	19(36.5)			
	>50	3(5.8)			
**Comorbidities ^3^**		22 (40.7)			
**Comorbidities ^3^**	Hypothyroidism	8 (14.8)			
	Hyperlipidemia	3 (5.6)			
	Diabetes	4 (7.4)			
	CVD	3 (5.6)			
	Other ^7^	3 (5.6)			
Treatment					
**Drug ^3^**	Dexamethasone	37 (33.6)			
	Remdesivir	30 (27.3)			
	Vitamin C	26 (23.6)			
	Vitamin D	23 (20.9)			
	Montelukast	11 (10.0)			
	Naproxen	15 (13.6)			
**Respiratory support status ^3^**	No Need	26 (54.2)			
	Nasal catheter	6 (12.5)			
	Oxygen mask	4 (8.3)			
	Reservoir bag	12 (25.0)			
**Hospitalization duration ^6^**		6.26 ± 2.58, (0,11)			

^1^Mean ± Standard deviation; ^2^Independent Samples t-Test; ^3^Frequency (Percent); ^4^Chi-squared test; ^5^One-way ANOVA; ^6^Mean ± Standard deviation, (minimum, maximum); ^7^Fatty liver, anxiety disorder, miscarriage, pregnancy, cardiac arrhythmia, asthma, allergy, angiography, mental disorder, dyslipidemia.

Out of the 54 patients, 36 (66.7%) had a fever, 32 (59.3%) had a cough, 20 (37.0%) experienced myalgia, and 28 (51.9%) reported chills. The most common antiviral drug used was remdesivir, administered to 30 patients (27.3%), while the most common anti-inflammatory drug was dexamethasone, prescribed to 37 patients (33.6%). In terms of respiratory support, 26 patients (54.2%) did not require any support, while 12 patients (25.0%) needed a reservoir bag, 6 patients (12.5%) required a nasal catheter, and 4 patients (8.3%) were provided with an oxygen mask.

**Figure 2:**
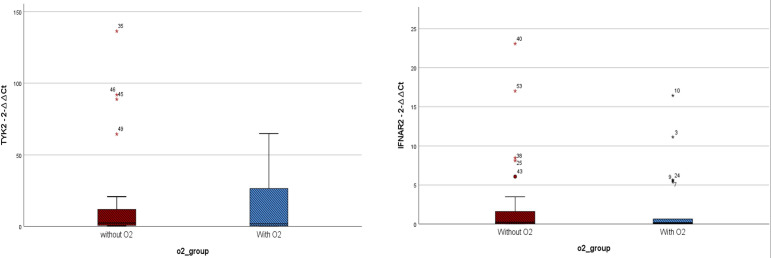
Expressions of IFNAR2 and TYK2 transcripts among COVID-19 patients who received oxygen(O2) therapy (n = 22) and those who did not require it (n = 27). TYK2 2^−ΔΔCt^ values in the patients who received O2 and the group that did not need O2 were 14.57 ± 21.85 and 17.55 ± 34.98, respectively. IFNAR2 2^−ΔΔCt^ values in the patients who received O2 and the group that did not need O2 were 2.13 ± 4.29 and 2.68 ± 5.61, respectively. A two-tailed t-test was used to compare between two groups. Mild outliers are marked with a circle (O) and extreme outliers are marked with a star (*).

**Figure 3:**
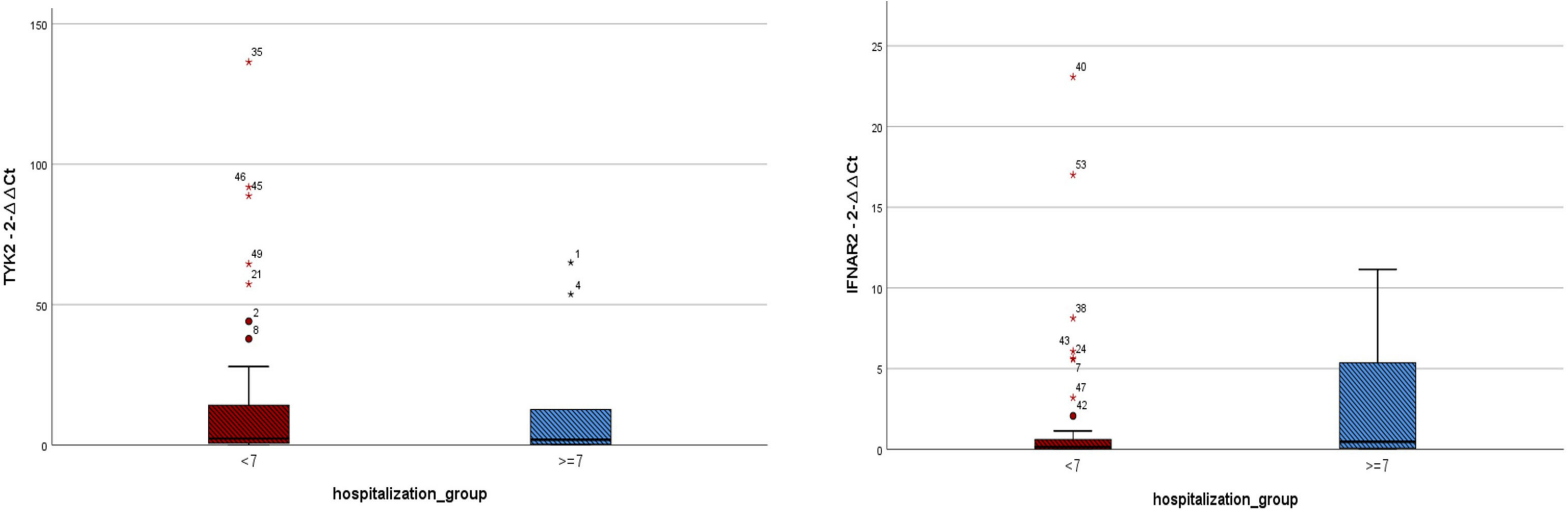
Expressions of IFNAR2 and TYK2 transcripts among COVID-19 patients hospitalized for less than 7 days (n = 41) and those hospitalized for ≥ 7 days (n = 10). TYK2 2^−ΔΔCt^ values in the patients with hospitalization duration < 7 days and those hospitalized for ≥ 7 days were 16.47 ± 30.38 and 14.12 ± 24.27, respectively. IFNAR2 2^−ΔΔCt^ values in the patients with hospitalization duration < 7 days and those hospitalized for ≥ 7 days were 1.86 ± 4.64 and 2.67 ± 4.13, respectively. A two-tailed t-test was used to compare between two groups. Mild outliers are marked with a circle (O) and extreme outliers are marked with a star (*).

The mean hospitalization duration was 6.26 ± 2.58 days, with a range of 0 to 11 days. Regarding gene expression levels, there was a negligible difference for both IFNAR2 and TYK2 between patients who received oxygen therapy and those who did not require it (*p*-value = 0.708 and *p*-value = 0.729, respectively, **Figure 2**). The level of expression in both the IFNAR2 and TYK2 genes did not significantly differ between patients hospitalized for less than 7 days and those hospitalized for ≥7 days (*p*-value = 0.618 and *p*-value = 0.822, respectively, **Figure 3**). We analyzed the correlation between the expression levels of IFNAR2 and TYK2 and demographic and clinical data. Our findings show a weak correlation between the expressions of IFNAR2 and CRP (*p*-value = 0.045, r = 0.192). After adjusting for potential confounding factors (age, sex, and history of COVID-19 vaccination injection), we observed a negative correlation between the expression levels of IFNAR2 and TYK2 transcripts in COVID-19 patients (*p*-value = 0.044; partial correlation coefficient = -0.283). However, in healthy individuals, after controlling for the same confounders, there was no significant correlation between the expression of IFNAR2 and TYK2 transcripts (*p*-value = 0.112; partial correlation coefficient = -0.221). The ΔCt and ΔΔCt for IFNAR2 were significantly higher in the case group compared to the control group [6.29 *vs*. 3.99, *p*-value = 0.002; 2.03 *vs*. 1 × 10^−15^, *p*-value = 0.002, respectively, (**Table 2**)], suggesting lower gene expression in the case group. The ΔCt and ΔΔCt levels for TYK2 were notably higher in the COVID-19 patients than in the controls [9.02 *vs*. 7.79, *p*-value = 0.028; 1.23 *vs*. 2.8571 × 10^−10^, *p*-value = 0.028, respectively, (**Table 2**)], suggesting decreased gene expression in the COVID-19 patients. However, there was no significant difference in gene expressions for both IFNAR2 and TYK2 among the subgroups of the case group (mild, moderate, and severe) (**Table 3**, *p*-value > 0.05). Our combined gene expression cluster analysis for IFNAR2 and TYK2 showed 2 clusters (**Table 4**). A linear regression analysis was conducted to predict hospitalization duration based on the IFNAR2 ΔΔCt and TYK2 ΔΔCt values. Surprisingly, the results indicated that neither the IFNAR2 ΔΔCt (B = -0.184, 95% CI: -0.524–0.157, *p*-value = 0.275) nor the TYK2 ΔΔCt (B = 0.114, 95% CI: -0.268–0.496, *p*-value = 0.543) were significant predictors of hospitalization duration.

**Table 2:** Comparison of ΔCt, ΔΔCt, and 2^−ΔΔCt^ between the case and control groups.

	Control	Case	*p*-Value
IFNAR2 ^1,3^ ΔCt	3.99 ± 3.56	6.29 ± 3.84	0.002 ^5^
TYK2 ^2,3^ ΔCt	7.79 ± 2.71	9.02 ± 3.07	0.028 ^5^
IFNAR2 ^1,3^ ΔΔCt	1 × 10^−15^ ± 3.56	2.30 ± 3.84	0.002 ^5^
TYK2 ^2,3^ ΔΔCt	2.8571 × 10^−10^ ± 2.71	1.23 ± 3.07	0.028 ^5^

^1^Interferon alpha and beta receptor subunit 2; ^2^Tyrosine kinase 2; ^3^Mean ± Standard deviation; ^5^Independent samples’ t-test.

**Table 3:** Comparison of ΔCt, ΔΔCt, and 2^−ΔΔCt^ among mild, moderate, and severe groups.

	Mild	Moderate	Severe	p-Value
TYK2 ^2,3^ ΔCt	9.04 ± 2.58	8.20 ± 3.29	9.63 ± 3.10	0.452 ^5^
IFNAR2 ^1,3^ ΔCt	6.39 ± 4.47	6.64 ± 3.02	5.43 ± 4.09	0.697 ^5^
TYK2 ^2,3^ ΔΔCt	1.97 ± 2.90	0.26 ± 3.04	1.62 ± 3.20	0.169 ^5^
IFNAR2 ^1,3^ ΔΔCt	1.91 ± 4.35	2.66 ± 3.01	1.37 ± 4.52	0.681 ^5^
TYK2 ^1,4^ 2^−ΔΔCt^	12.93 ± 30.94, 1.14	9.38 ± 17.31, 0.63	13.49 ± 17.53, 6.10	0.451 ^6^
IFNAR2 ^2,4^ 2^−ΔΔCt^	3.24 ± 5.99, 0.28	0.88 ± 2.08, 0.17	4.27 ± 6.33, 0.39	0.712 ^6^

^1^Interferon alpha and beta receptor subunit 2; ^2^Tyrosine kinase 2; ^3^Mean ± Standard deviation; ^4^Mean ± Standard deviation, Median; ^5^One-way ANOVA; ^6^Kruskal–Wallis.

**Table 4:** Combined gene expression cluster analysis of ΔΔCt of both IFNAR2 and TYK2.

Cluster	Number of subjects in each cluster	Cluster Centers^3^	
		IFNAR2^1^ ΔΔCt	TYK2^2^ ΔΔCt
1	61	3.97	2.39
2	47	-2.46	-1.58

^1^Interferon alpha and beta receptor subunit 2; ^2^Tyrosine kinase 2; ^3^K-means clustering.

**Figure 4:**
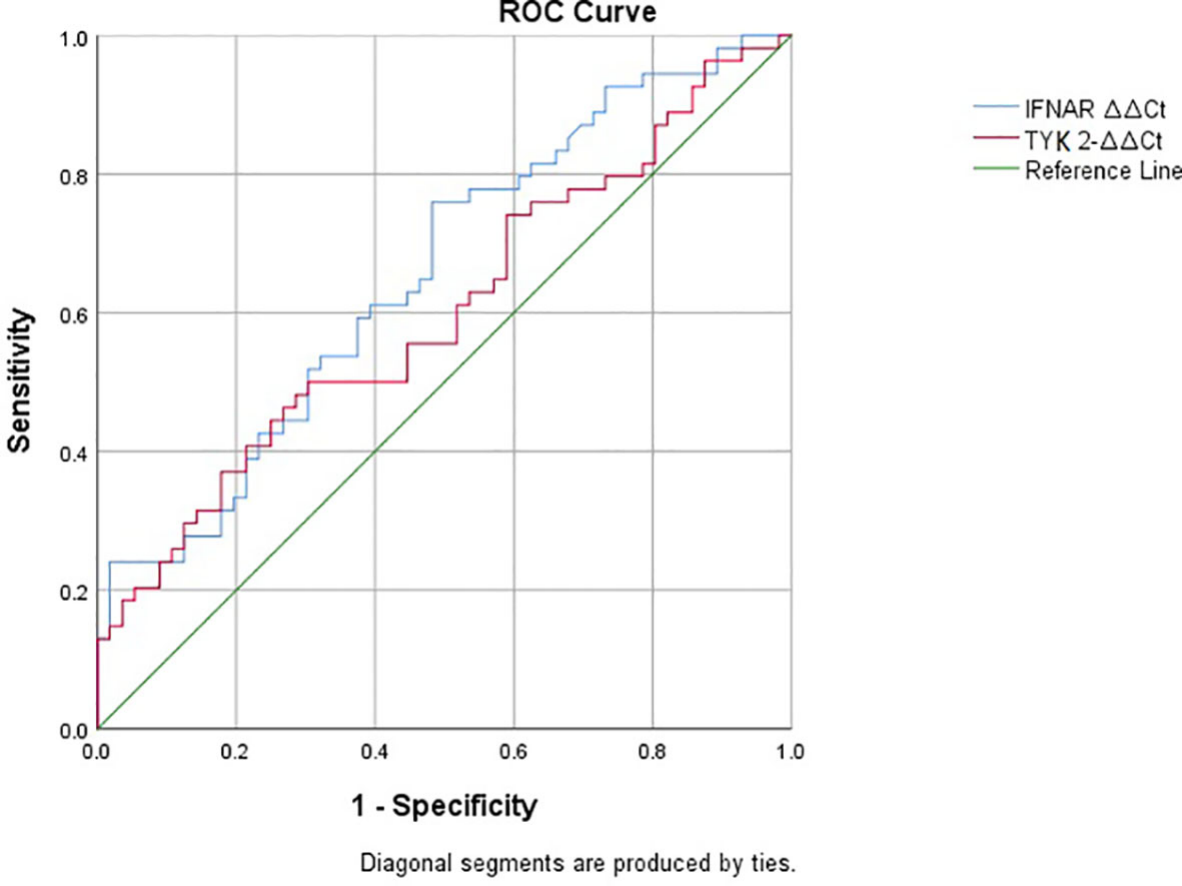
ROC curves showing the prognostic power of IFNAR2 and TYK2 expressions in COVID-19.

The ROC curve for IFNAR2 ΔΔCt showed an area under the curve (AUC) of 0.655 (*p*-value = 0.005, 95% CI: 0.554–0.757, **Figure 4**). In contrast, the ROC curve for TYK2 ΔΔCt yielded an AUC of 0.602, which was not statistically significant (*p*-value = 0.066, 95% CI: 0.495–0.708, **Figure 4**). The IFNAR2 ΔΔCt = −0.21 demonstrated a sensitivity of 0.75 and specificity of 0.51. Moreover, the TYK2 ΔΔCt = −0.126 showed a sensitivity of 0.55 and a specificity of 0.55. Additionally, ROC analysis was repeated based on the level of CRP among two groups of patients with positive and negative CRP levels. ROC curve assessed prognostic power of TYK2 ΔΔCt in patients with negative CRP showed an AUC of 0.340 (*p*-value=0.356, CI: 0.000-0.763). Also, it showed an AUC of 0.781 (*p*-value=0.025, CI: 0.619-0.943) for patients with positive levels of CRP.

## 4 Discussion

The global impact of the COVID-19 pandemic since 2019 has resulted in significant morbidity and mortality worldwide (Ciotti et al., 2020). Extensive research has investigated the interplay between genetic factors and COVID-19 susceptibility and severity. Among the key determinants of disease progression, cytokines play a crucial role, and variations in these factors are closely linked to the severity and outcomes of COVID-19 cases (Akbari et al., 2020). The heightened susceptibility of certain individuals to severe respiratory infections has been distinctly associated with specific genetic variants. Host genetic variants have been linked to disruptions in vital immune response proteins during COVID-19 pathogenesis (Choudhary et al., 2021). These disruptions can lead to severe symptoms in susceptible individuals. Our study explores the possible role of two genes in shaping COVID-19 prognosis.

Genome-wide association studies (GWASs) focusing on SARS-CoV-2 infection dynamics have significantly illuminated the potential involvement of IFNAR2 and TYK2 (Ma et al., 2021; Aghamirli and Saleh-Gohari, 2023). A higher expression of IFNAR2 was linked to a reduced probability of critical COVID-19, and TYK2 expression had a role in regulating IFNAR signaling (Velavan et al., 2021). Our findings also demonstrate a significant reduction in the expression levels of IFNAR2 and TYK2 among COVID-19 patients. Despite the recognized role of IFNs in reducing viral replication through IFN receptor signaling, it is perplexing that severe COVID-19 cases exhibit significantly lower IFN levels compared to other viral infections (Hadjadj et al., 2020). This scenario could underscore the strategies employed by coronaviruses, which are adept at concealing viral RNA from pattern recognition receptors, allowing for covert replication. These viruses bind to specific proteins that hinder IFN responses, evade detection, or directly inhibit IFNAR signaling (Acharya et al., 2020). TYK2, a crucial cytokine receptor-associated kinase, notably emerges as an essential for IFNAR signaling in this regulatory network (Velavan et al., 2021). These genes have also gained clinical interest as potential targets for COVID-19 treatment. Several clinical trials have investigated the role of glucocorticoids in treating COVID-19 at its early stages, and glucocorticoids are capable of inhibiting IFNAR signaling. On the other hand, baricitinib is a Janus kinase (JAK) 1/2 inhibitor with the potential to inhibit TYK2. Both baricitinib and tocilizumab are recommended for COVID-19 treatment. Recent meta-analyses indicate no difference in mortality reduction between the two drugs; however, adverse reactions following baricitinib might be significantly fewer than tocilizumab (Zhang et al., 2023; Albuquerque et al., 2023). However, this evidence contradicts the findings of the study by Akbari et al., where no significant difference in IFNAR2 expression was observed between the controls and cases, and TYK2 expression was only significantly lower in males (Akbari et al., 2022). In the study by Akbari et al., the reported AUC was 0.53 for IFNAR1 and 0.58 for TYK2, resulting in a combined AUC of 0.57 for both genes, which did not reach statistical significance. The contrasting findings may be attributed to variations in sampling methodologies and laboratory protocols. Furthermore, various conditions and factors, including diseases and medications, have the potential to influence IFNAR2 and TYK2 expressions. Additionally, a limited sample size might have had an impact on these findings and further studies with a larger population would yield different values of AUCs.

Evidence highlighting the significance of IFNAR2’s role in COVID-19 severity spans various studies, ranging from genetic and transcriptional levels to soluble protein dynamics. Associations between the IFNAR2 locus and COVID-19 severity have been identified through various GWAS and multi-omic analyses (Akter et al., 2022). Despite ongoing controversies, the interaction between various cytokines, including IFN, and their impact on COVID-19 severity and clinical outcomes remains apparent. Fricke-Galindo et al. reported a connection between reduced IFNAR2 expression and severe COVID-19 in their study (Fricke-Galindo et al., 2022). This observation is corroborated by the notable decrease in sIFNAR2 levels among non-survivors. Patients who did not require invasive mechanical ventilation had higher sIFNAR2 levels compared to those who did, and the sIFNAR2 levels showed a weak correlation with the duration of invasive mechanical ventilation. Another study examining IFNAR2 single-nucleotide variants revealed their association with clinical outcomes, particularly in severe cases. Survivors and non-survivors exhibited distinct expression profiles (Dieter et al., 2022). The recent study by Yaugel-Novoa reported no significant difference between IFNAR1 concentrations among COVID-19 groups based on severity; however, the IFNAR2 concentration was significantly related to more severe COVID-19. This article suggests that some of the discrepancies among different studies might be attributed to the difference between isoforms of IFNAR2 as it has been suggested that the soluble isoform is linked to impairment of the immune system in combating the coronavirus through IFNAR signaling (Yaugel-Novoa et al., 2023). Our study reveals no significant difference in IFNAR2 and TYK2 expressions among three COVID-19 subgroups, including mild, moderate, and severe COVID-19. We were focused on IFNAR2 expression, and measuring IFNAR2 isoforms was not our objective. Future research focusing on post-transcriptional modifications in IFNAR signaling could fill current gaps and also hints at research on IFNAR2 expression among COVID-19 patients. The small number of cases and controls might have had an impact on the significance level resulting in our study. Despite discrepancies in findings regarding the downregulation of IFNAR2 and TYK2 in COVID-19 patients, the importance of these genes in the pathogenesis of COVID-19 remains prominent.

The expressions of both IFNAR2 and TYK2 showed no significant association with the need for oxygen therapy or hospitalization exceeding seven days. It is worth noting that the correlation between IFNAR2 expression and CRP levels appeared to be relatively weak. However, the reported AUCs in our study (0.65 for IFNAR2 and 0.60 for TYK2) indicated statistical significance, but only for IFNAR2. It also should be noted that despite this significant *p*-value, the AUCs in our study were not at the level to conclude the strong prognostic role of these genes. Therefore, the results of this study need to be interpreted cautiously regarding poor discrimination of AUC values due to the small sample size. The findings by Akbari et al. showed no significant difference in the expression levels of IFNAR1 and TYK2 between individuals requiring ICU admission due to severe COVID-19 and those who did not. Although the AUCs for IFNAR1 and TYK2 were reported as 0.49 and 0.59, respectively, the combined analysis resulted in an AUC of 0.57, but none of these reached statistical significance (Akbari et al., 2022). Another study by Zabihi Rizi et al. demonstrated an association between TYK2 single-nucleotide variants and COVID-19 severity, as indicated by significant outcomes in ROC analysis (Zabihi Rizi et al., 2023). In a large-scale GWAS conducted by Pairo-Castineira et al., utilizing Mendelian randomization methods, they found that a low expression of IFNAR2 and a high expression of TYK2 both contribute to the severity of COVID-19 (Pairo-Castineira et al., 2021). While our findings align with the studies mentioned, they do not support the up-regulation of TYK2. This controversy could be attributed to the difference in sample size and ethnicity between populations. However, impaired IFNAR signaling and low IFNAR2 expression appear to be related to the severity of COVID-19, as supported by various studies. These findings highlight the importance of IFNAR2 expression both in the complex puzzle of COVID-19 pathogenesis and also in the clinical context to understand its possible prognosis and potential therapeutic implications.

Based on our findings, the negative correlation between IFNAR2 and TYK2 expression levels, even after adjusting for confounding factors, highlights their potential role in COVID-19 pathogenesis. These genes are likely involved in similar biological pathways or regulatory networks. This discovery from the current study warrants investigation within the JAK-STAT signaling pathway.

We showed that both IFNAR2 ΔΔCt and TYK2 ΔΔCt are insignificant predictors of hospitalization duration. In other words, changes in these gene expression levels do not reliably predict how long a patient with COVID-19 will be hospitalized. These findings suggest that other factors beyond IFNAR2 and TYK2 expression may be important in determining hospitalization duration for COVID-19 patients.

To determine whether CRP can impact the prognosis of COVID-19 in conjunction with the expression of two specific genes, we separated the study participants into two groups - one with positive CRP levels and one with negative CRP levels, based on the reference laboratory. Although the AUC of TYK2 ΔΔCt was 0.781 in positive CRP subjects and 0.340 in negative CRP subjects, there was no significant difference due to overlapping confidence intervals. Some findings from a subgroup analysis of Wuhan research showed CRP can be considered a dependable predictor of adverse outcomes in COVID-19 patients with different disease severity (AUC 0.832, z=10.23, p<0.001; AUC 0.989, z=44.04, p<0.001). On admission, CRP was a strong predictor of severe/critical illness (AUC 0.783, z=10.69, p<0.001) (Luo et al., 2020). The discrepancy in the results can be attributed to differences in the study design and statistical analysis methods used.

While our study yields significant findings regarding COVID-19 prognosis, it is essential to acknowledge a few limitations. Despite diligent efforts to minimize confounding factors, fully eliminating them proved to be a challenging task. While our stringent inclusion criteria were designed to enroll cases and controls without underlying diseases or drug consumption, the prevailing circumstances of the Omicron pandemic required us to consider individuals with prior infection or vaccination. Additionally, the study population was limited. Despite precisely calculating the number of cases and controls based on appropriate statistical methods, conducting large-scale studies could bring a better understanding of these genes’ prognostic roles. Furthermore, the absence of a follow-up phase and the lack of survival analysis represent additional potential limitations. Other studies might consider studying the dynamic changes of gene expression across severity or intervention using longitudinal samples. Also, conducting studies to investigate the loss of function mutations in these genes or studies at the protein level would be an interesting topic for future investigators since our objective was limited to the RNA level. Nevertheless, this study makes a valuable contribution to our understanding of COVID-19 pathogenesis, shedding light on the genetic factors that significantly influence prognosis.

## 5 Conclusions

This study determines that there were no significant gene expression differences for IFNAR2 and TYK2 among patients requiring oxygen therapy or differing hospitalization durations. A significant finding emerged when comparing COVID-19 patients with controls, which demonstrated lower gene expressions of IFNAR2 and TYK2 in COVID-19 cases. However, when conducting an analysis of different severity subgroups within the cases, no significant gene expression variations were observed. Importantly, the analysis of the study produced a significant AUC value of 0.655 for IFNAR2 expression, signifying its poor discriminative value, and TYK2 expression’s AUC of 0.602 did not reach statistical significance.

## Data availability statement

The original contributions presented in the study are included in the article/**Supplementary Material**. Further inquiries can be directed to the corresponding author.

## Ethics statement

The studies involving humans were approved by Ethics Committee of Mazandaran University of Medical Sciences (IR.MAZUMS.REC.1400.10406). The studies were conducted in accordance with the local legislation and institutional requirements. The human samples used in this study were acquired from primarily isolated as part of your previous study for which ethical approval was obtained. Written informed consent for participation was not required from the participants or the participants’ legal guardians/next of kin in accordance with the national legislation and institutional requirements. Written informed consent has been obtained from the patient(s) to publish this paper.

## Author contributions

AR: Conceptualization, Formal analysis, Investigation, Methodology, Supervision, Writing – original draft, Writing – review & editing. MR: Conceptualization, Investigation, Writing – original draft. YH: Investigation, Writing – original draft. GC: Investigation, Writing – original draft. YG: Investigation, Writing – original draft. RG: Investigation, Writing – original draft. RA-N: Conceptualization, Formal analysis, Investigation, Supervision, Writing – review & editing. HY: Investigation, Writing – original draft, Writing – review & editing. MS: Writing – review & editing. MT: Formal analysis, Writing – original draft. RV: Investigation, Writing – original draft. FM: Investigation, Writing – original draft. AT: Conceptualization, Investigation, Supervision, Writing – review & editing. BR: Investigation, Writing – original draft.

## References

[B1] Acharya D. Liu G. Gack M. U. (2020). Dysregulation of type I interferon responses in COVID-19. Nat. Rev. Immunol. 20, 397–398. doi: 10.1038/s41577-020-0346-x 32457522 PMC7249038

[B2] Aghamirli S. S. Saleh-Gohari N. (2023). ACE2, TMPRSS2, TYK2, SLC6A20, and IFNAR2 human genes variants influence SARS−CoV−2 infection susceptibility. Authorea Preprints doi: 10.22541/au.169035663.39913026/v1

[B3] Akbari H. Tabrizi R. Lankarani K. B. Aria H. Vakili S. Asadian F.. (2020). The role of cytokine profile and lymphocyte subsets in the severity of coronavirus disease 2019 (COVID-19): a systematic review and meta-analysis. Life Sci. 258, 118167. doi: 10.1016/j.lfs.2020.118167 32735885 PMC7387997

[B4] Akbari M. Akhavan-Bahabadi M. Shafigh N. Taheriazam A. Hussen B. M. Sayad A.. (2022). Expression analysis of IFNAR1 and TYK2 transcripts in COVID-19 patients. Cytokine 153, 155849. doi: 10.1016/j.cyto.2022.155849 35339044 PMC8894869

[B5] Akter S. Roy A. S. Tonmoy M. I. Q. Islam M. S. (2022). Deleterious single nucleotide polymorphisms (SNPs) of human IFNAR2 gene facilitate COVID-19 severity in patients: a comprehensive in silico approach. J. Biomol. Struct. Dyn. 40, 11173–11189. doi: 10.1080/07391102.2021.1957714 34355676

[B6] Albuquerque A. M. Eckert I. Tramujas L. Butler-Laporte G. McDonald E. G. Brophy J. M.. (2023). Effect of tocilizumab, sarilumab, and baricitinib on mortality among patients hospitalized for COVID-19 treated with corticosteroids: a systematic review and meta-analysis. Clin. Microbiol. Infect. 29, 13–21. doi: 10.1016/j.cmi.2022.07.008 35863630 PMC9293401

[B7] Available online at: https://www.covid19treatmentguidelines.nih.gov/overview/clinical-spectrum.

[B8] Baillie J. K. (2014). Targeting the host immune response to fight infection. Science 344, 807–808. doi: 10.1126/science.1255074 24855243

[B9] Blanco-Melo D. Nilsson-Payant B. E. Liu W.-C. Uhl S. Hoagland D. Møller R.. (2020). Imbalanced host response to SARS-CoV-2 drives development of COVID-19. Cell 181, 1036–45.e9. doi: 10.1016/j.cell.2020.04.026 32416070 PMC7227586

[B10] Chan Y. (2003). Biostatistics 104: correlational analysis. Singapore Med. J. 44, 614–619. doi: 10.12691/ajmsm-5-4-3 14770254

[B11] Choudhary S. Sreenivasulu K. Mitra P. Misra S. Sharma P. (2021). Role of genetic variants and gene expression in the susceptibility and severity of COVID-19. Ann. Lab. Med. 41, 129. doi: 10.3343/alm.2021.41.2.129 33063674 PMC7591285

[B12] Ciotti M. Ciccozzi M. Terrinoni A. Jiang W.-C. Wang C.-B. Bernardini S. (2020). The COVID-19 pandemic. Crit. Rev. Clin. Lab. Sci. 57, 365–388. doi: 10.1080/10408363.2020.1783198 32645276

[B13] Dieter C. de Almeida Brondani L. Lemos N. E. Schaeffer A. F. Zanotto C. Ramos D. T.. (2022). Polymorphisms in ACE1, TMPRSS2, IFIH1, IFNAR2, and TYK2 genes are associated with worse clinical outcomes in COVID-19. Genes 14, 29. doi: 10.3390/genes14010029 36672770 PMC9858252

[B14] Fitzgerald K. A. McWhirter S. M. Faia K. L. Rowe D. C. Latz E. Golenbock D. T.. (2003). IKKϵ and TBK1 are essential components of the IRF3 signaling pathway. Nat. Immunol. 4, 491–496. doi: 10.1038/ni921 12692549

[B15] Fricke-Galindo I. Martínez-Morales A. Chávez-Galán L. Ocaña-Guzmán R. Buendía-Roldán I. Pérez-Rubio G.. (2022). IFNAR2 relevance in the clinical outcome of individuals with severe COVID-19. Front. Immunol. 13, 949413. doi: 10.3389/fimmu.2022.949413 35967349 PMC9374460

[B16] Gallo Marin B. Aghagoli G. Lavine K. Yang L. Siff E. J. Chiang S. S.. (2021). Predictors of COVID-19 severity: a literature review. Rev. Med. Virol. 31, 1–10. doi: 10.1002/rmv.2146 PMC7855377 32845042

[B17] Hadjadj J. Yatim N. Barnabei L. Corneau A. Boussier J. Smith N.. (2020). Impaired type I interferon activity and inflammatory responses in severe COVID-19 patients. Science 369, 718–724. doi: 10.1126/science.abc6027 32661059 PMC7402632

[B18] Johnson H. M. Lewin A. S. Ahmed C. M. (2020). SOCS, intrinsic virulence factors, and treatment of COVID-19. Front. Immunol. 11, 582102. doi: 10.3389/fimmu.2020.582102 33193390 PMC7644869

[B19] Kaser A. (2020). Genetic risk of severe Covid-19. Mass Med. Soc; p, 1590–1591. doi: 10.1056/NEJMe2025501 PMC7583681 33053291

[B20] Kawasaki T. Kawai T. (2014). Toll-like receptor signaling pathways. Front. Immunol. 5, 461. doi: 10.3389/fimmu.2014.00461 25309543 PMC4174766

[B21] Low Z. Y. Wen Yip A. J. Chow V. T. Lal S. K. (2022). The Suppressor of Cytokine Signalling family of proteins and their potential impact on COVID-19 disease progression. Rev. Med. Virol. 32, e2300. doi: 10.1002/rmv.2300 34546610 PMC8646547

[B22] Luo X. Zhou W. Yan X. Guo T. Wang B. Xia H.. (2020). Prognostic value of C-reactive protein in patients with coronavirus 2019. Clin. Infect. Dis. 71, 2174–2179. doi: 10.1093/cid/ciaa641 32445579 PMC7314209

[B23] Ma Y. Huang Y. Zhao S. Yao Y. Zhang Y. Qu J.. (2021). Integrative genomics analysis reveals a 21q22. 11 locus contributing risk to COVID-19. Hum. Mol. Genet. 30, 1247–1258. doi: 10.1093/hmg/ddab125 33949668 PMC8136003

[B24] Pairo-Castineira E. Clohisey S. Klaric L. Bretherick A. D. Rawlik K. Pasko D.. (2021). Genetic mechanisms of critical illness in COVID-19. Nature 591, 92–98. doi: 10.1038/s41586-020-03065-y 33307546

[B25] Rahimi F. Abadi A. T. B. (2020). Practical strategies against the novel coronavirus and COVID-19—the imminent global threat. Arch. Med. Res. 51, 280–281. doi: 10.1016/j.arcmed.2020.03.005 32229157 PMC7270650

[B26] Shojaei M. Shamshirian A. Monkman J. Grice L. Tran M. Tan C. W.. (2023). IFI27 transcription is an early predictor for COVID-19 outcomes, a multi-cohort observational study. Front. Immunol. 13, 1060438. doi: 10.3389/fimmu.2022.1060438 36685600 PMC9850159

[B27] Thiel V. Weber F. (2008). Interferon and cytokine responses to SARS-coronavirus infection. Cytokine Growth Factor Rev. 19, 121–132. doi: 10.1016/j.cytogfr.2008.01.001 18321765 PMC7108449

[B28] Velavan T. P. Pallerla S. R. Rüter J. Augustin Y. Kremsner P. G. Krishna S.. (2021). Host genetic factors determining COVID-19 susceptibility and severity. EBioMedicine 72, 103629. doi: 10.1016/j.ebiom.2021.103629 34655949 PMC8512556

[B29] Wallweber H. J. Tam C. Franke Y. Starovasnik M. A. Lupardus P. J. (2014). Structural basis of recognition of interferon-α receptor by tyrosine kinase 2. Nat. Struct. Mol. Biol. 21, 443–448. doi: 10.1038/nsmb.2807 24704786 PMC4161281

[B30] Yaugel-Novoa M. Bourlet T. Longet S. Botelho-Nevers E. Paul S. (2023). Association of IFNAR1 and IFNAR2 with COVID-19 severity. Lancet Microbe. 6, e487 doi: 10.1016/S2666-5247(23)00095-2 PMC10072862 37028439

[B31] Zabihi Rizi F. Ghorbani A. Zahtab P. Darbaghshahi N. N. Ataee N. Pourhamzeh P.. (2023). TYK2 single-nucleotide variants associated with the severity of COVID-19 disease. Arch. Virol. 168, 119. doi: 10.1007/s00705-023-05729-2 36959416 PMC10035968

[B32] Zeberg H. Pääbo S. (2020). The major genetic risk factor for severe COVID-19 is inherited from Neanderthals. Nature 587, 610–612. doi: 10.1038/s41586-020-2818-3 32998156

[B33] Zhang J. Fan X. Zhang X. Jiang F. Wu Y. Yang B.. Efficacy and safety of tocilizumab and baricitinib among patients hospitalized for COVID-19: a systematic review and meta-analysis. Front. Pharmacol. (2023) 14, 1293331. doi: 10.3389/fphar.2023.1293331 38074144 PMC10703388

[B34] Zhang Q. Bastard P. Cobat A. Casanova J.-L. (2022). Human genetic and immunological determinants of critical COVID-19 pneumonia. Nature 603, 587–598. doi: 10.1038/s41586-022-04447-0 35090163 PMC8957595

