# Supplementary material for: Evaluation of IFNAR2 and TYK2 transcripts’ prognostic role in COVID-19 patients: a retrospective study
Source: Front Cell Infect Microbiol. 2024 Apr 29;14:1356542. doi: 10.3389/fcimb.2024.1356542 (PMC11089198; doi:10.3389/fcimb.2024.1356542)
Supplement: Supplementary file 1 [file DataSheet_1.docx]

Supplementary Material

# Supplementary Data

None.

# Supplementary Figures and Tables

## Supplementary Figures

*Validating and visualizing the results obtained from real-time PCR by gel electrophoresis*


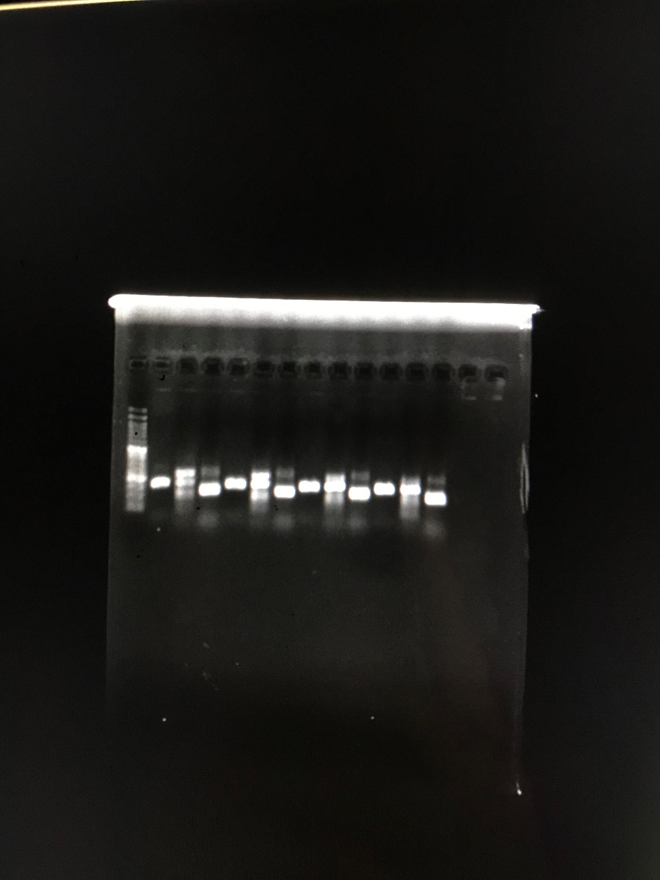


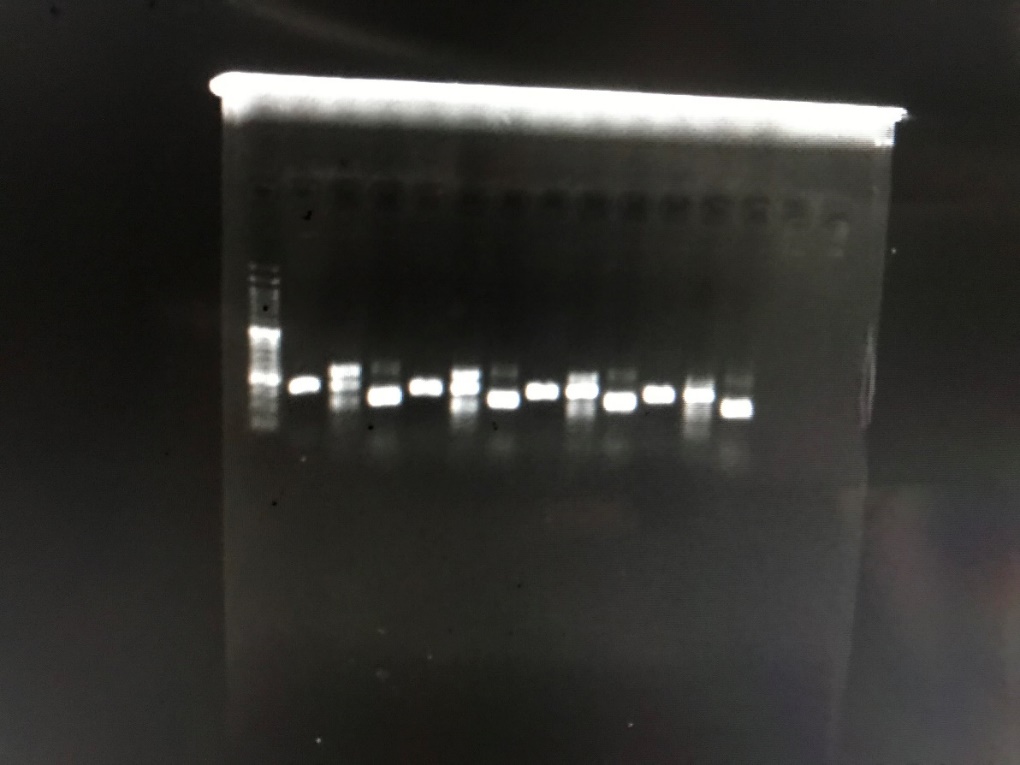


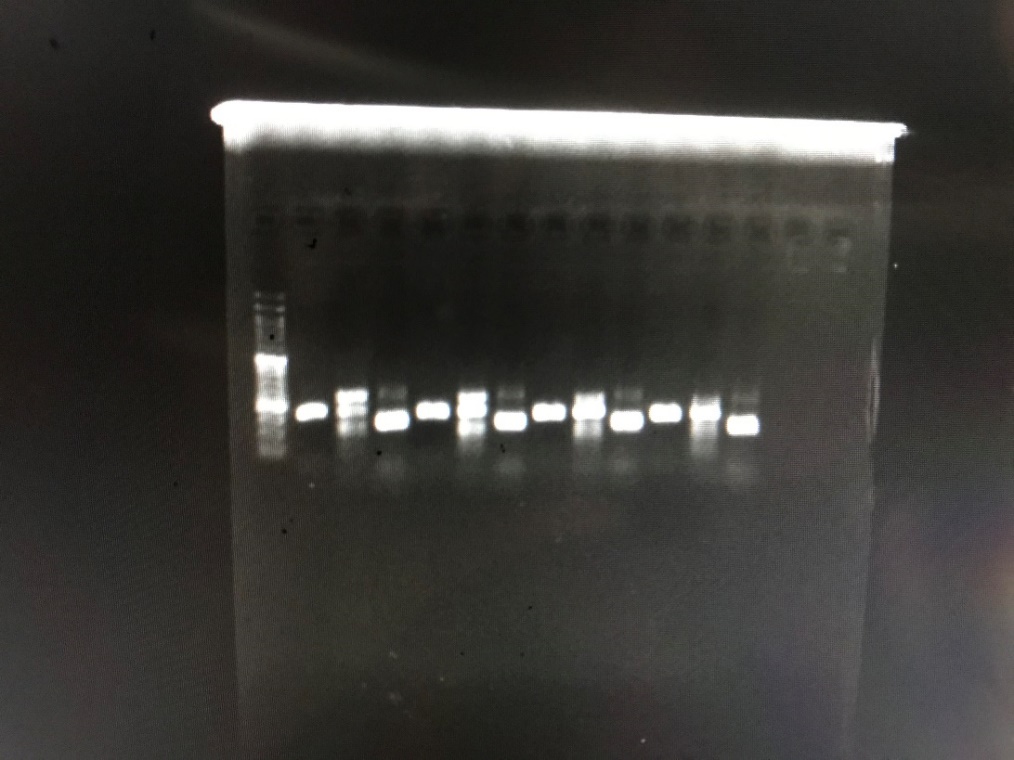


## Supplementary Tables

**Supplementary Table 1.** Complete laboratory results

|  | Mean ± Standard deviation (minimum, maximum) |
| --- | --- |
| SPO2 (%) | 95.6852±3.54924, (85,100) |
| CRP (mg/dL) | 21.12±29.712, (1,84) |
| ESR | 20.4±18.645, (2,79) |
| D-Dimer ng/ml | 760.924±1099.9998, (135,5790) |
| CPK | 341.76±352.864, (45,1212) |
| LDH (IU/L) | 468.44±264.252, (196,1285) |
| IL-6 (pg/ml) | 8.617±2.7672, (5.2,12) |
| AST | 42.13±17.546, (16,92) |
| ALT | 35.65±22.373, (8,99) |
| ALP | 183.41±75.31, (109,458) |
| Calcium | 8.796±0.4903, (7.9,9.6) |
| Magnesium | 2.354±0.5816, (1.5,4.1) |
| Phosphorus | 3.867±0.9508, (2.3,6.1) |
| Albumin | 4.442±0.4462, (3.8,5.4) |
| Urea | 29.13±11.653, (10,69) |
| Creatinine | 1.039±0.3222, (0.7,2.5) |
| Na | 130.083±34.9832, (4.2,145) |
| K | 13.448±35.058, (3.3,142) |
| Bilirubin T | 0.7374±0.51283, (0.2,2.33) |
| Bilirubin D | 0.257±0.1369, (0.08,0.66) |
| BS | 141.04±44.364, (89,237) |
| PH | 7.3735±0.05149, (7.28,7.47) |
| HCO3 | 24.391±2.8783, (19.2,30.5) |
| sO2c | 36.587±26.5114, (2.3,86.7) |
| pCO2 | 42.97±6.4306, (32.1,58.1) |
| BE-ECF | -0.265±2.926, (-6.3,5.1) |
| pO2 | 22.7±13.134, (3,52) |
| WBC count | 6462.55±2171.54, (3000,14700) |
| Lymphocyte (%) | 30.085±11.746, (5,60) |
| Lymphocyte count | 1910.54±967.521, (516,8131) |
| Neutrophil (%) | 61.212±13.8195, (32,90.9) |
| Neutrophil count | 4081.94±2051.772, (1530,13362) |
| Mix (%) | 9.186±6.4803, (1,33.6) |
| Mix | 581.38±440.401, (60,2567) |
| Platelets | 228354.55±58680.067, (133000,378000) |
| Hemoglobin | 13.348±1.8405, (7.4,18) |

**Supplementary Table 2.** Symptoms

|  | Frequency (N, %) |
| --- | --- |
| Fever | 36 (66.7%) |
| Cough | 32 (59.3%) |
| Dyspnea | 5 (9.3%) |
| Headache | 17 (31.5%) |
| Sore throat | 9 (16.7%) |
| Fatigue | 8 (14.8%) |
| Myalgia | 20 (37.0%) |
| Chills | 28 (51.9%) |
| Lethargy | 11 (20.4%) |
| Anorexia | 5 (9.3%) |
| Dyspnea | 6 (11.1%) |
| Chest pain | 6 (11.1%) |
| Rhinorrhea | 6 (11.1%) |

**N:** Number, **%:** Percent
